# Seroinfection of Antibodies to *Toxoplasma gondii*, Parvovirus B19, *Treponema pallidum*, and HIV in a Pregnant Attending a Medical Center in Northern Peru

**DOI:** 10.1155/2024/8844325

**Published:** 2024-05-27

**Authors:** Deniss Cubas-Alarcón, Génesis Masiel Guevara-Vásquez, Danny Omar Suclupe-Campos, Salvadora Castro-Martínez, Franklin Rómulo Aguilar-Gamboa, Virgilio E. Failoc-Rojas

**Affiliations:** ^1^ Microbiology Laboratory Hospital Regional Docente Belén, Lambayeque, Peru; ^2^ Research Management Hospital Regional Lambayeque, Lambayeque, Peru; ^3^ Microbiology Laboratory Clinical Laboratory Service School of Biological Sciences Universidad Nacional Pedro Ruiz Gallo, Lambayeque, Peru; ^4^ Clinical Laboratory Centro de salud de Motupe, Lambayeque, Peru; ^5^ Immunology and Virology Laboratory Hospital Regional de Lambayeque, Lambayeque, Peru; ^6^ Grupo de investigación en Inmunología y Virología del Norte, Lambayeque, Peru; ^7^ Universidad San Ignacio de Loyola, Lima, Peru

## Abstract

**Introduction:**

Transplacental infections are frequent, especially in developing countries, where limited screening is performed to find infectious agents in the pregnant population. We aim to determine the clinical and epidemiological characteristics and seroinfection of antibodies against *Toxoplasma*, parvovirus B19, *T. pallidum*, and HIV in pregnant women who attended the Motupe Health Center in Lambayeque, Peru during July-August 2018.

**Methods:**

A descriptive cross-sectional study was conducted in 179 pregnant women interviewed with a standardized questionnaire. ELISA was used to determine antibodies to *Toxoplasma* and parvovirus B19. The detection of syphilis and HIV was conducted using immunochromatography, while the detection of hepatitis B was conducted using FTA-ABS and immunofluorescence, respectively.

**Results:**

Of 179 pregnant women, syphilis and HIV infections routinely included in the screening of pregnant women presented a seroinfection of 2.2 and 0.6%, respectively. Toxoplasmosis seroinfection was 25.1%, while IgM antiparvovirus B19 was 40.8%, revealing that pregnant women had an active infection at the time of study.

**Conclusion:**

The level of seroinfection of toxoplasmosis reveals the risk to which pregnant women who participated in the study are exposed. The high seroinfection of parvovirus B19 could explain the cases of spontaneous abortion and levels of anemia in newborn that have been reported in Motupe, Lambayeque, Peru. However, future causality studies are necessary to determine the significance of these findings.

## 1. Introduction

Vertical transmission of infectious diseases continues to be a major public health problem in contemporary Latin America, with rates that can reach 28% [1]. These infections are classified according to their origin as transplacental infections, perinatal infections, and postpartum infections [2]. Among these, transplacental infections specifically affect prenatal development and occur during pregnancy through the hematogenous spread of pathogenic agents that reach both the placenta and the fetus [2]. Infectious agents transmitted exclusively through this route include *Toxoplasma gondii* (*T. gondii*), parvovirus B19 (B19V), and rubella virus (RV), while other agents, such as *Treponema pallidum*, cytomegalovirus (CMV), HIV, herpes simplex virus (HSV), varicella-zoster virus (VZV), and more recently Zika virus (ZIKV) [3], can also be transmitted through this route, though not exclusively [2].

Epidemiological studies conducted throughout the world show high seroprevalence rates of the etiological agents involved in transplacental infections. Asian countries report prevalences of *T. gondii* antibodies ranging from 30.7% to 31.7% in pregnant women [4, 5]. In African countries, seroprevalence rates of 0.9% to 3.9% for syphilis and 2.0% to 10.33% for HIV have been observed among pregnant women in rural areas [6–8]. Several studies have found a significant prevalence of *T. gondii* infection among pregnant women, reaching 51% [9], and a 74.3% association between *T. gondii* infection and HIV patients [10]. Additionally, a study conducted in Tanzania revealed that more than half of pregnant women have IgG antibodies against B19V, with approximately one-third of them assessing positive for IgM [11].

The seroprevalence of these infections varies widely in Latin American countries [12]. Reports from Mexico and Brazil indicate a prevalence of IgG antibodies against B19V among women of childbearing age of 50.1% and 31.1%, respectively [12]. In Brazil, the prevalence of IgG antibodies against *Toxoplasma gondii* in pregnant women is high, as proved by Câmara et al. [13] and Gontijo et al. [14], who reported rates of 77% and 68.3%, respectively.

In the health center of the Motupe district, Lambayeque, Peru, there are frequent laboratory reports of syphilis and HIV in pregnant women, which are diagnosed and confirmed by the Regional Health Management. Since 2008, it has been recognized that the integration and management of prenatal care with the HIV and syphilis detection and clinical management in this region face barriers such as geographical accessibility. This lack of accessibility leads to a higher number of maternal deaths, as the population lacks the financial means to travel [15]. Furthermore, syphilis, toxoplasmosis, and/or HIV can increase the risk of miscarriages [16, 17] as they can cross the placental barrier and infect embryonic tissues [18]. The occurrence of spontaneous abortions diagnosed solely by clinical evaluation, coupled with the frequent reporting of neonatal anemia, has led to the undertaking of the present research. The aim of this study is to determine the clinical and epidemiological characteristics, as well as the presence of antibodies against *T. gondii*, B19V, *T. pallidum*, and HIV in pregnant patients who attended the health center of the Motupe district in Lambayeque, Peru during the months of July-August 2018.

## 2. Methods

### 2.1. Design and Population

A descriptive cross-sectional study was conducted in a population consisting of pregnant patients who attended the Motupe Health Center in the department of Lambayeque, Peru, from July to August 2018.

To calculate the sample size, a seroprevalence proportion of 0.14 was expected for a finite population (6000; source “Censo Instituto Nacional De Estadistica”, 2017. Available at: https://munimotupe.gob.pe/wp-content/uploads/2020/11/PEI-2019-2021.pdf). Using the Epidat 4.2 program, a sample size of 179 pregnant patients was obtained. The sampling method used was systematic probabilistic sampling, starting with a random number and selecting participants in blocks. The inclusion criteria encompassed all pregnant women who attended the laboratory service for their first or second pregnancy control, whether they had comprehensive health insurance, and signed an informed consent form. Pregnant women who did not respond to some questions due to discomfort or those who did not complete the interview were excluded from the study.

### 2.2. Study Variables

The following variables were used: age—the sample was divided into the following age groups: 12-16, 17-21, 22-26, 27-31, 32-36, and 37 or older; marital status—single, married, or cohabiting; educational level—elementary, secondary, or higher education; place of origin—rural or urban; occupation—homemaker, student, or employed; gestational period—first, second, or third trimester; history of abortion—coded dichotomously, with “yes” indicating a history of abortion and “no” indicating no history; age of first menstruation—11-13, 14-16, or 17-19 years; and age of sexual debut—10-14, 15-19, or 20-24 years.

### 2.3. Procedures/Data Collection and Management

The recruitment procedure for the participants was started at the gynecology-obstetrics service of the Motupe Health Center, where the researcher (obstetrician) explained the aim of the study. Upon obtaining their consent, pregnant women were escorted to the health facility's clinical laboratory service, where a second member of the research team (biologist) conducted a standardized questionnaire interview to collect clinical and epidemiological data, including age, educational level, marital status, place of origin, occupation, gestational period, history of abortions, age of first menstruation, and age of sexual debut. Subsequently, samples were collected for serology studies, and the results were provided during the subsequent appointment in the gynecology-obstetrics service. The samples were transported to the Immunology and Virology Laboratory of the Research Directorate of the Lambayeque Regional Hospital (HRL) in two appropriately labeled containers, one primary and one secondary, and placed in a shipping package in accordance with the regulations specified in the Guide on Regulations for the Transport of Infectious Substances 2019-2020 [19].

For serological determination of the infectious agents under investigation, the immunochromatography technique was used for syphilis and HIV, following the instructions provided by the brand of ABON Biopharm product. These tests were carried out at the Motupe Health Center. Samples that assessed positive for syphilis were confirmed using the latex agglutination assay from the SPINREACT brand and sent to the Regional Health Management for further confirmation using the FTA-ABS technique. Similarly, samples that were evaluated positive for HIV were sent to the Regional Health Management for confirmation using immunofluorescence.

For the determination of antibodies against *T. gondii* and B19V, the enzyme-linked immunosorbent assay (ELISA) was used according to the manufacturer's instructions for the *VIRION SERION and RIDA SCREEN* kits, respectively. Both assays were performed in the immunology and virology area of the HRL.

### 2.4. Statistical Analysis

Statistical analysis was performed using STATA v.16.1 software (StataCorp LP, College Station, TX, USA). Absolute and relative frequencies of the clinical and epidemiological characteristics and seroprevalence of the variables studied were calculated. Additionally, the association between variables was explored using the chi-square test or Fisher's exact test as appropriate, considering a 95% confidence level and a significance level of *p* < 0.05.

A simple regression analysis was performed on the results of *T. gondii* and parvovirus B19 infection. However, a regression analysis for *T. pallidum* and HIV could not be performed due to the small number of results found. In the simple regression analysis, prevalence ratios (PR) and 95% confidence intervals (95% CI) were estimated using generalized linear regression models with a Poisson distribution family, a logarithmic link function, and robust variance. A multiple analysis was not performed due to the absence of association in the simple regression model.

### 2.5. Ethics Statement

The present research was approved by the Ethics Committee of the Regional Hospital of Lambayeque (0214-044-18), and informed consent was requested from participants. In the case of pregnant minors, the purpose of the study was explained to the pregnant woman, parents, and/or partner and was recorded in the informed consent form. In the case of pregnant women over 18 years of age, the study was explained to the pregnant woman and her partner, asking for the consent of the pregnant woman.

Codes were used to maintain the confidentiality of the data of patients eligible for this study. The ethical principles of the Declaration of Helsinki were respected.

## 3. Results

Of the 179 pregnant women who participated in the study, the majority (67.6%) were between 17 and 26 years old. 93.9% of them were homemakers, and 38% regularly attended the health center during the first months of pregnancy. The analysis of other variables is shown in Table 1.

Regarding the seroprevalence of antibodies, it was found that 45 (25.1%) out of the 179 pregnant women in the study were positive for IgG antibodies against *T. gondii*, but no positivity was observed for IgM antibodies. On the other hand, 73 (40.8%) out of 179 pregnant women showed IgM antibodies against B19V. Additionally, 4 (2.2%) pregnant women were identified with syphilis, and one (0.6%) with HIV infection (see Table 2).

Regarding the exploration of pregnant women's characteristics based on seroprevalence, it was found that the age extremes, specifically 12 to 16 years and 32 years and above, had the highest frequency of seropositive patients for *T. gondii* and parvovirus B19. A lower frequency of women with higher education was observed in relation to *T. gondii* compared to those with primary education (13.2% vs. 30.4%, respectively), with their association showing marginal significance in the regression analysis (OR: 0.43; 95% CI: 0.17-1.09). However, this difference was not observed for parvovirus. Being from a rural area was more often associated with either of the two seroprevalences, resulting in a 30% decrease in the frequency of *T. gondii* and a 35% decrease in the frequency of parvovirus B19 among those from urban areas.

Women who started sexual activity at an early age (10 to 14 years) had a higher frequency of *T. gondii* (50.0% vs. 25.0%) and parvovirus (66.7% vs. 35.0%) compared to those who initiated sexual activity after the age of 20. In other words, starting sexual activity at 20 years or later decreased the frequency of *T. gondii* and parvovirus by 50% (95% CI: 0.12-2.09) and 47% (95% CI: 0.23-1.20), respectively. Please refer to Table 3 for further details.

No statistical association was found between clinical and epidemiological characteristics and toxoplasmosis or parvovirus infection (Table 4).

## 4. Discussion

This research found that 25.1% of pregnant women had IgG antibodies against *T. gondii*, while none of the pregnant women showed positive results for IgM. This outcome suggests that the pregnant women did not have an active infection during the study. However, the frequency of IgG in this group reveals earlier exposure to infection and may incur a risk during pregnancy because they do not have protective antibodies against the parasite that would protect them from possible complications in the event of acquiring it, especially in the first months of pregnancy. However, approximately one-third of the global population is seropositive for *T. gondii*, and the disease is acquired by consuming unwashed vegetables and fruits containing the parasite's oocysts [20]. Furthermore, it has been shown that consuming undercooked meat and encountering infected cat feces are significant risk factors for infection [20, 21]. A study conducted in the same region reported a seroprevalence of 35.8% for *T. gondii* [22], which aligns with the findings of this research. Cultural and sociodemographic factors may also influence these findings, as proved in a report from São Paulo, Brazil [23], where 62% and 3.4% of 574 pregnant women tested positive for IgG and IgM anti-*T. gondii*, respectively, a rate that is double what was reported in this study.

Reports from Western countries show that 50% of pregnant women have evidence of past B19V infection [24]. Furthermore, it is known that acute infection in pregnant women ranges from 1% to 2%, and during epidemic periods, it can exceed 10% [25]. In our study, the seroprevalence of IgM antibodies against B19V in pregnant women was 40.8%. This finding corresponds to the report by Mirambo et al. in Tanzania [11], who reported a seroprevalence of IgM and IgM/IgG anti-B19V of 32.8% and 19.4% in patients within the first three months of gestation, and the study by Warnecke et al. [12], where the seroprevalence of antibodies against B19V in women of childbearing age ranged from 36% to 55% in Brazil, Mexico, Germany, Poland, Turkey, and China. Although a limitation of this research was the absence of IgG anti-B19V testing, the seroprevalence IgM revealed that B19V was the primary etiological agent to which pregnant women were exposed.

In pregnant women, transplacental transmission of B19V is a potential risk to the fetus, as it infects erythroid progenitor cells in the bone marrow and liver, blocking fetal erythropoiesis and causing severe anemia, hydrops, and/or fetal death [26]. Neonatal anemia is a common event in Peru [27] and is recognized as a public health problem, particularly in children under the age of three, with potential long-term consequences for child development [28]. In 2018, the Lambayeque region in Peru reached a 41.0% prevalence of anemia in children under 36 months, one of the highest rates in its history [29]. Determining the high exposure of pregnant women to B19V could play a significant role in understanding the development of this event, a fact that, based on the findings of this study, deserves to be considered and further investigated.

More than a million people are estimated to contract sexually transmitted infections (STIs) every day [30]. Syphilis and HIV are the most common STIs, and coinfection is common [31]. 1.8 million pregnant women worldwide are estimated to be infected with syphilis, and less than 10% are diagnosed and treated [32]. Congenital syphilis has two modes of transmission—transplacental and during childbirth—and it is recommended to diagnose it at the beginning of prenatal care [33]. In our study, the seroprevalence of syphilis in pregnant women was 2.2%. Related results have been found in other regions of Peru, where prevalences of 1.08 [34] and 2.49% [35] have been reported, exceeding the national level reported in 2017, which was 0.3% [36]. This finding shows that congenital syphilis remains a public health problem in the Americas region, and although Peru is among the countries that have made progress, the elimination goal was not achieved [37].

HIV infection during pregnancy is associated with severe consequences for the mother, fetus, and newborn, such as maternal death, abortion, stillbirth, and low birth weight [38]. In the present study, a seroprevalence of 0.6% was detected in pregnant women, which is consistent with other studies and indicates that it is not an emerging problem in this population [39, 40]. No variable considered in clinical and epidemiological characteristics was significantly associated with the seropositivity of any pathogen, with a *p*-value >0.05. However, it was seen that pregnant women between 17 and 26 years of age had the highest levels of infection, both for B19V and other pathogens. A considerable proportion of the studied population completed secondary education or higher. This study has provided important data on the incidence of this infection, standing for the starting point for future studies.

The American College of Obstetricians and Gynecologists [41] notes that maternal infections can be transmitted to the child, causing congenital infections. Congenital infections such as cytomegalovirus (CMV), parvovirus B19, varicella-zoster virus (VZV), and toxoplasmosis can cause moderate to severe complications in the fetus and infant. For pregnant women, it is recommended to perform evaluations using IgG avidity assays for CMV and toxoplasmosis. If a woman has low avidity IgG and positive IgM, it is likely that she has recently acquired the infection. Additionally, PCR testing in amniotic fluid is recommended for the diagnosis of fetal CMV infection, ideally after 21 weeks of gestation. In cases of suspected parvovirus exposure, screening tests for B19, IgG, and IgM are recommended. If the woman's IgM titer against parvovirus is positive, she is presumed to be infected, and fetal infection may occur. According to the World Health Organization, it is estimated that there are nearly 2 million stillbirths annually. The incidence of stillbirth is estimated at 77 cases per 1000 live births. In a study from Italy [42], the most frequent causes of stillbirth were nulliparity, advanced maternal age, and coagulative disorders, with no cases found due to TORCH infections or parvovirus. However, it is known that many pregnant women who have TORCH or parvovirus during pregnancy can end up with fetal death. In the United States of America [43], the lethality rate for newborn who contracted syphilis was found to be 30%. The global loss (combined fetal and infant losses per 100,000 registrable births) was estimated at 139.6 (95% CI, 130.9-148.3) for any infectious cause and 15.2 (95% CI, 12.3-18.1) for viral infections, and more than a third (37%) of the deaths attributed to viruses were before live births, due to parvovirus (63%) or cytomegalovirus (33%). Cytomegalovirus was associated with a global loss rate of 3.1 (95% CI, 1.8-4.4) and an infant mortality rate of 1.3 (95% CI, 0.4-2.1) per 100,000 live births; 91% of the cases were congenital infections [44].

A meta-analysis found that viral infections early in pregnancy increase the risk of congenital heart diseases [45]. Therefore, pregnant women with acute infections are recommended to undergo echocardiography to assess potential problem [41, 46].

### 4.1. Limitations and Strengths

An important limitation of this study is the lack of a temporal relationship due to its cross-sectional design, which makes it impossible to establish a causal relationship between *T. gondii* and parvovirus infection. Furthermore, since sociodemographic variables were collected through self-reports, there was the possibility of response bias. The assessment of risk factors was based on the responses of participants, which introduces measurement bias. Finally, the study was limited to a specific region of Peru, so the results cannot be extrapolated to all pregnant Peruvian women. However, the study highlights a potential problem in Peru, providing useful information for future research and public health policies in the region.

Furthermore, future research and programs must be developed to fully inform patients, family members, and physicians about the risks of stillbirth in the case of congenital infections during pregnancy to prevent primary infection [47, 48].

## 5. Conclusions

Pregnant women attending the Motupe, Lambayeque, Peru, showed moderate levels of exposure to pathogens associated with transplacental infections. Understanding the epidemiology of these infections helps us to better understand their dynamics in the pregnant population, as well as the knowledge of the microorganisms involved. No variable considered in clinical and epidemiological characteristics was significantly associated with the seropositivity of any pathogen. The low seroprevalence of IgG anti-*T. gondii* indicates the risk to which pregnant women are exposed. On the other hand, the high seroprevalence of B19V could explain the reported cases of spontaneous abortion and neonatal anemia in Motupe. However, causal studies are necessary to determine the significance of these findings.

## Figures and Tables

**Table 1:** Clinical and epidemiological characteristics of pregnant women who attended at the health center of Motupe, Peru.

Variables	*N* = 179	%
*Age*		
12-16 years	8	4.5
17-21 years	66	36.9
22-26 years	55	30.7
27-31 years	28	15.6
32 years or more	22	12.3
*Educational level*		
Primary	46	25.7
Secondary	95	53.0
Higher	38	21.2
*Civil status*		
Single	14	7.8
Married	16	8.9
Cohabiting	149	83.2
*Place of origin*		
Rural	101	56.4
Urban	78	43.6
*Occupation*		
Homemaker	168	93.8
Student/worker	11	6.2
*Gestational period*		
1st trimester	68	38.0
2nd trimester	69	38.6
3rd trimester	42	23.5
*Abortion*		
No	162	90.5
Yes	17	9.5
*Menarche*		
11-13 years	138	77.1
14-19 years	41	23.9
*Age of sexual initiation*		
10-14 years	6	3.5
15-19 years	153	85.5
20-24 years	20	11.2

**Table 2:** Seroprevalence of antibodies in pregnant women who attended at the health center of Motupe, Peru.

Variables	*N* = 179	%
*Toxoplasma gondii IgG*		
Negative	134	74.9
Positive	45	25.1
*Parvovirus B19 IgM*		
Negative	106	59.2
Positive	73	40.8
*HIV*		
Negative	178	99.4
Positive	1	0.6
*Treponema pallidum*		
Negative	175	97.8
Positive	4	2.2

IgM: immunoglobulin M; IgG: immunoglobulin G; HIV: human immunodeficiency virus.

**Table 3:** Clinical-epidemiological characteristics according to the results of antibody serofrequency in pregnant women who attended at the health center of Motupe, Peru.

Variables	*Toxoplasma gondii*			Parvovirus B19		
	Positive (%)	Negative (%)	*p* value	Positive (%)	Negative (%)	*p* value
*Age*						
12-16 years	3 (37.5)	5 (62.5)	0.412	4 (50.0)	4 (50.0)	0.719
17-21 years	17 (25.8)	49 (74.2)		23 (34.8)	43 (65.2)	
22-26 years	13 (23.6)	42 (76.4)		23 (41.8)	32 (58.2)	
27-31 years	4 (14.3)	24 (85.7)		12 (42.9)	16 (57.1)	
32 years or more	8 (36.4)	14 (63.6)		11 (50.0)	11 (50.0)	
*Educational level*						
Primary	14 (30.4)	32 (69.6)	0.147	20 (43.5)	26 (56.5)	0.910
Secondary	26 (27.4)	69 (72.6)		38 (40.0)	57 (60.0)	
Higher	5 (13.2)	33 (86.8)		15 (39.5)	23 (60.5)	
*Civil status*						
Single	3 (21.4)	11 (78.6)	0.429	3 (21.4)	11 (78.6)	0.249
Married	2 (12.5)	14 (87.5)		8 (80.0)	8 (50.0)	
Cohabiting	40 (26.9)	109 (73.1)		62 (41.6)	87 (58.4)	
*Place of origin*						
Rural	29 (28.7)	72 (71.3)	0.210	44 (43.6)	57 (56.4)	0.389
Urban	16 (20.5)	62 (79.5)		29 (37.2)	49 (62.8)	
*Occupation*						
Homemaker	43 (25.6)	125 (74.4)	0.583	70 (41.7)	98 (58.3)	0.347
Student/worker	2 (18.2)	9 (81.8)		3 (27.3)	8 (72.7)	
*Gestational period*						
1st trimester	16 (23.5)	52 (76.5)	0.842	32 (47.1)	36 (52.9)	0.400
2nd trimester	19 (27.5)	50 (72.5)		26 (37.7)	43 (62.3)	
3rd trimester	10 (23.8)	32 (76.2)		15 (35.7)	27 (64.3)	
*Abortion*						
No	39 (24.1)	123 (75.9)	0.310	67 (41.4)	95 (58.6)	0.628
Yes	6 (35.3)	11 (64.7)		6 (35.3)	11 (64.7)	
*Menarche*						
11-13 years	34 (24.6)	104 (75.4)	0.776	56 (40.6)	82 (59.4)	0.919
14-19 years	11 (26.8)	36 (73.2)		17 (41.5)	24 (58.5)	
*Age of sexual initiation*						
10-14 years	3 (50.0)	3 (50.0)	0.360	4 (66.7)	2 (33.3)	0.378
15-19 years	37 (24.2)	116 (75.8)		62 (40.5)	91 (59.5)	
20-24 years	5 (25.0)	15 (75.0)		7 (35.0)	13 (65.0)	

*p* value is obtained from the chi-square test.

**Table 4:** Association of clinical and epidemiological characteristics according to the results of antibody serofrequency in pregnant women who attended at the health center of Motupe, Peru.

Variables	*Toxoplasma gondii*			Parvovirus B19		
	RP	IC 95%	*p* value	RP	IC 95%	*p* value
*Age*						
12-16 years	Ref.			Ref.		
17-21 years	0.69	0.26-1.84	0.456	0.70	0.32-1.50	0.358
22-26 years	0.63	0.23-1.74	0.373	0.84	0.39-1.79	0.646
27-31 years	0.38	0.11-1.37	0.139	0.86	0.38-1.94	0.711
32 years or more	0.97	0.34-2.78	0.954	1.00	0.44-2.25	1.00
*Educational level*						
Primary	Ref.			Ref.		
Secondary	0.90	0.52-1.55	0.704	0.92	0.61-1.39	0.692
Higher	0.43	0.17-1.09	0.077	0.91	0.54-1.52	0.713
*Civil status*						
Single	Ref.			Ref.		
Married	0.58	0.25-1.37	0.555	2.33	0.62-8.80	0.138
Cohabiting	1.25	1.15-1.36	0.707	1.94	0.61-6.19	0.204
*Place of origin*						
Rural	Ref.			Ref.		
Urban	0.71	0.39-1.32	0.280	0.85	0.53-1.36	0.395
*Occupation*						
Homemaker	Ref.			Ref.		
Student/worker	0.71	0.17-2.93	0.636	0.65	0.21-2.08	0.399
*Gestational period*						
1st trimester	Ref.			Ref.		
2nd trimester	1.17	0.60-2.27	0.643	0.80	0.48-1.34	0.271
3rd trimester	1.02	0.46-2.23	0.977	0.76	0.41-1.46	0.259
*Abortion*						
No	Ref.			Ref.		
Yes	1.47	0.62-3.46	0.383	0.85	0.44-1.67	0.643
*Menarche*						
11-13 years	Ref.			Ref.		
14-19 years	1.09	0.55-2.15	0.806	0.919	0.67-1.54	0.919
*Age of sexual initiation*						
10-14 years	Ref.			Ref.		
15-19 years	0.48	0.15-1.57	0.226	0.61	0.33-1.11	0.103
20-24 years	0.50	0.12-2.09	0.343	0.53	0.23-1.20	0.126

Regression generalized linear models with Poisson family, logistic link. PR: prevalence Ratio; CI 95%: confidence interval at 95%; Ref: reference.

## Data Availability

The data will be available upon reasonable request.
